# Removal of the Uterine Appendages

**Published:** 1886-06

**Authors:** J. Greig Smith

**Affiliations:** Surgeon to the Bristol Royal Infirmary


THE BRISTOL

### Medico-Chirurgical Journal.

JUNE, 1886.

### REMOVAL OF THE UTERINE APPENDAGES.

BY

J. Greig Smith,

*Surgeon to the Bristol Royal Infirmary.*

*(Continued from page 27.)*

*The Operation.*—What will be the exact nature and extent of the operation depends partly on the disease for which the operation is performed, and partly on the end which the operator seeks to secure.

In diseases purely local and one-sided, such as ovarian hernia, single hydrosalpinx, simple ovarian abscess, uncomplicated prolapse of one ovary, and many examples of Fallopian pregnancy, there need be no hesitation in leaving the sound organs untouched. In double hydrosalpinx without adhesions, the ovaries may be left behind. But in all cases where nerve disturbances have existed for some time, we must reckon with the force of diseased habit; and here complete delivery from pain may be secured by nothing short of complete abrogation of function. If a patient had been suffering for years from a small abscess in one ovary, the appendages being otherwise normal, her cure would almost certainly be more speedy and more complete if the whole of the appendages were swept away. Each case must be judged on its own merits. The duration of the disease and the extent of it; the age of the patient; her position in life; her own and her husband's wishes, must all be taken into account.

It happens in most cases of inflammatory disease, that the lesions are bilateral; and here the question is settled for us. In those cases where the motive is abrogation of function, or cessation of uterine bleeding, we have to decide between simple removal of the ovaries,—oöphorectomy, and removal of both tubes and ovaries,—salpingo-oöphorectomy.

Into a discussion of the influence which the tubes may possess over the function of menstruation we cannot here enter. Suffice it to say, that if Tait's theories have not everywhere been admitted, his practice has been very generally followed. The modern operation is removal of the appendages, and not merely removal of the ovaries.

Practical considerations in support of this practice need only be given here. A few of these are as follows:

(1) More than one case has been recorded in which removal of tubes without ovaries has been followed by menopause. One such case has occurred in my own practice. In this case menopause was not sought. On the other hand, many cases of double oöphorectomy have not been followed by complete change of life. The best results published so far have been got from complete removal of the appendages.

(2) A better pedicle is afforded for deligation when both tubes and ovaries are included. A ligature surrounding the ovarian attachments alone is liable to cause kinking of the tube with subsequent risk of occlusion or distension. The hilum of the ovary, with its plexus of vessels, is not satisfactory tissue for inclusion in ligature.

(3) By removal of the tubes all further risks of disease in them are done away with. The ovaries can scarcely be removed without upsetting the blood supply to the tubes, and causing some physical injury to them as well. Venous congestion or actual inflammation in the tubes might readily follow simple oöphorectomy, and nullify the benefits of the operation.

(4) The tubes are useless when the ovaries are removed. If one good reason for their removal can be given, and none for their being left behind, the question at issue is settled.

Removal of the uterine appendages may be either a very simple or a very difficult proceeding. If the organs occupy their normal situations and there are no adhesions, as in the cases to which the term castration may with least impropriety be applied, for small bleeding myomata, or absent or malformed uterus, or cystic ovaries, or such like, the operation is easy. If, as in inflammatory disease, they are displaced and matted together and to the surrounding organs, the operation may be one of great difficulty and delicacy. For large myomata, again, the operation presents special features which may cause difficulties; and, finally, the proceeding in the case of ovarian hernia is peculiar to itself.

*Where the Appendages are Anatomically Normal, or nearly so.*—The incision, made in the ordinary median position between the umbilicus and pubes, need not be longer than an inch and a half or two inches—enough easily to admit two fingers. At the second or third cut the fibres of the linea alba are laid bare through the whole length of the wound. Pressure forceps are placed on any bleeding points, and left attached. As the parietes are not thinned and distended by a tumour, the linea alba is very narrow, and it is not often that it can be divided without exposing one or both recti. A small opening is made in the fascia: if it is on the linea alba, well and good; if not, the layers are pushed to one side or the other, and when the situation of the fibrous septum is found, the fascia is slit up to the length of the wound by the point of the knife cutting forwards. The muscular fibres are pushed to one side with the handle of the scalpel, and the sub-peritoneal fat exposed. This is caught up on two catch forceps, and carefully divided between them: and the rest of the division is made while the tissues are pulled out of the wound. The peritoneum is easily recognised: a small opening is made in it while it is thus everted; the finger inserted into this opening acts as a director upon which the division is completed, preferably by scissors. By this method, which is Tait's, there is no danger in wounding bowels; as each fold of tissue is pulled up and made tense it is cut on its folded edge by the blade of the knife, held horizontally: and when the very smallest opening has been made in the peritoneum the air rushes in, and the bowels, if they have been dragged forward by suction, fall back at once. With moderate care, there is not the slightest danger of wounding bowel: it is idle to use it as an argument against the operation, as one distinguished gynæcologist has done, that there is great danger of wounding the bowel.

The compression forceps may now be removed. It is sometimes advisable, if the peritoneum is loosely attached, to leave a catch forceps on each side clinging to its cut margin, and leave these lying over the abdominal walls.

The two first fingers are now inserted into the wound. If omentum covers the bowels, it must be dragged upwards; if not, the fingers are pushed straight down to the fundus uteri. The fingers, one on each side of the broad ligament, and grasping it between them, are now carried outwards till the ovary is felt; it is then lifted out of the wound, with its mesovarium and its oviduct. Still held in this position in the left hand, the Fallopian tube is pulled out as much as it will readily come, and the pedicle spread out for ligature. The parts to be removed are the ovary with its mesovarium, and the Fallopian tube in its outer three-fourths, with the double peritoneal fold in which it lies, and which contains also the parovarium and the vascular erectile tissue known as the bulb of the ovary. The ligature is placed double by transfixing with a blunt needle. The inner pedicle contains the utero-ovarian ligament, the Fallopian tube somewhere near its isthmus, the spermatic artery and its veins, and the small branch which accompanies the Fallopian tube. The outer ligature lies at the retiring angle where the infundibulo-pelvic and infundibulo-ovarian ligaments meet, takes its half of the mesovarium, and also constricts the spermatic artery. In most cases no method of ligature is, in my opinion, superior to Tait's Staffordshire knot. The loop carried through by the needle (which must be very blunt, to prevent the accident of wounding any of the numerous small veins) is caught by a finger and the needle withdrawn. The loop is then raised over the parts to be removed, and one of the free ends drawn through the loop and over it. The free ends are now caught in one hand and pulled tight, while the fingers and thumb of the other hand act as an opposing force at the site of deligation. The knot is then cast and tied tightly, either by the operator unaided, or with the help of the assistant, who pulls on one end of the ligature. The parts are then cut away, by successive snips of scissors, at a distance of about a quarter of an inch from the ligature. Before making the last cut the surface must be carefully inspected, to see that there is no bleeding. The pedicle is then dropped in, and a soft round sponge is put in after it, so as to make sure, from the absence of blood on it, that the ligation is perfect. A more deliberate, and perhaps more clumsy, plan is, to catch the sides of the pedicle in fixation forceps, hand these over to an assistant, and apply the ligature below them. The same proceeding is then carried out with the appendages on the opposite side, and a sponge placed on the pedicle. This is essentially Tait's method of operating: it is the best with which I am acquainted.

A small thin flat sponge is now placed over the bowels under the incision; and the sutures, four or five in number, are introduced. For this purpose I prefer using a Goetz's suture-instrument, with a needle which I have had specially made, to all other methods. The instrument is essentially a handled needle, in the handle of which is kept a reel of suture silk soaking in antiseptic lotion. As the sutures are introduced they are cut off in convenient lengths. Each suture includes skin, muscle, fascia, and peritoneum. When the sutures are placed, the sponges are removed by two fingers inserted into the abdomen, and if they return free of blood the wound is closed.

*When the Appendages are Inflamed and Adherent.*—The previous operation is a very simple one. From first to last, in competent hands, it can scarcely occupy more than fifteen minutes. But it is a very different thing if the appendages are adherent or inflamed, or suppurating and matted together, as in inflammatory diseases. Then the operation may be one of the most difficult in surgery. Even in the hands of surgeons of the highest skill, it has not unfrequently been abandoned as impracticable.

The first difficulty met with is, probably, that the appendages are fixed and cannot be drawn to the surface. They may be represented by an irregular conglomeration of cystic and cicatricial material sessile on the broad ligament or in Douglas's pouch, and perhaps intimately adherent to bowels. They are beyond the reach of sight, however much the abdominal walls are depressed. To deal with such a state of affairs, one of two courses is open. The first is to enlarge the incision to three or four inches; to pull the bowels out of the pelvis and keep them in the abdomen by one or more sponges packed under them; to pull the parietes apart by spatulæ, and seek by a strong light to expose the parts to view, and operate by the aid of sight. This may be safe, but it is clumsy and difficult. If the parietes are muscular and firm, considerable force may be required to crowd the bowels into the abdomen, and to keep them there is still more difficult. And it is not easy at the bottom of the pelvis to perform delicate surgical manipulations with knife or scissors and ligature. One operator has had recourse to the doubtful expedient of making space by turning the bowels outside the abdomen altogether.

The other course is that followed by Tait. As a result of his unrivalled experience, Tait has come to the conclusion that it is best to depend entirely on the fingers to deal with such a condition, relying on the skilled sense of touch to guide against the dangers of tearing bowel or other structures. To control bleeding he recommends sponge packing. Firstly, the fingers map out the actual limits of the diseased organs; then these are gently separated from all surrounding organs, and gradually the mass is unfolded upwards from behind till the only attachment left is the proper pedicle of the parts to be removed. Even as thus separated the appendages will probably be found sessile on the broad ligament, so that they can be little more than brought within the range of sight. The broad ligaments are stretched tightly across the pelvis, and dragging on the appendages may tear them. The pedicle ligature may have to be carried under the diseased parts at a considerable depth from the surface. If possible, all the tissues are gathered together in one pedicle, as by the Staffordshire knot; but the puckering so produced may drag upon the opposite ligament so much as to cause tearing. To tie in two parts almost of necessity tears open the tissue between them. It has happened to me in one case, while putting on a ligature, that the broad ligament was torn clean away from the side of the uterus for a distance of more than an inch.

In dealing with such a difficulty, Tait, always fertile in expedients, tells me that he is in the habit of pushing his finger down on the broad ligament, close to the pelvic insertion of it, and so causing a series of minute tears through the fibrous fasciæ and peritoneum, but leaving the elastic, distensile, and tortuous vessels uninjured.

I have thought that an air bag inflated in the rectum might sometimes be of use by raising the whole pelvic floor.

The bleeding in these cases is sometimes described as being truly alarming, and I have had practical experience of this fact. Sponges are packed in everywhere as the adhesions are separated, and as the hæmorrhage is started. If, after the appendages have been removed, bleeding still goes on, a little solution of iodine on a sponge may be applied to the raw surfaces. Of course visible bleeding points are dealt with by ligature or forci-pressure. And it may sometimes be good practice to leave forceps attached to bleeding points for twenty-fours hours or so, their handles being left outside. In all such cases the insertion of a glass drainage-tube for a day or two is advisable.

If an abscess or abscesses exist, extra care is necessary to avoid rupture of the abscess wall. It may be wise before beginning separation to aspirate the contents, and place a pressure forceps on the opening so made. In such cases the placing of sponges all round the diseased parts is peculiarly necessary.

*For Uterine Myoma.*—For small myomata the proceeding may be in no way different from the simplest operation. In fact, as the appendages are raised with the fundus, and the broad ligaments are usually soft and distensile, the operation may be easier.

When the tumour is large, and especially when it is adherent, the difficulties may be great, even insuperable. Not a few such operations, begun as oöphorectomy, have to be finished as hysterectomy. If the tumour grows away from the uterus, being sub-peritoneal and near the fundus, the appendages may be deep in the pelvis. Where the growth lies between the broad ligaments the ovaries will be elevated, and squeezed between the tumour and the parietes. In an unsymmetrical growth one ovary may be quite conveniently near the surface, while the other lies out of reach and behind. Indeed, we must expect an endless variety of situation, and in some cases be prepared not to find ovaries at all.

When one ovary is found, we must, before proceeding to remove it, find the other: and before removing one we must be certain that it is possible to remove both. Having decided to remove the appendages, we rotate the tumour to one side, so as to bring the parts first to be removed as close as possible to the surface. The pedicle is secured in the ordinary way by a Staffordshire knot, or in any other way that seems suitable for the case. Thornton's plan, of not cutting off the first ovary till all manipulations are over with the second, is a good one: it minimises the risk of bleeding from the divided pedicle by disturbing it. Forceps are left attached to the appendages first ligated: the appendages on the other side are brought as near the surface as possible by rotating the growth, tied, cut off, and covered with a flat sponge. The appendages on the other side are then cut off, and also covered with a sponge. When the sutures have been inserted in the parietal wound, the sponges are removed and the wound closed.

*For Ovarian Hernia.*—When, from pain or symptoms of strangulation, it has become necessary to operate upon an ovarian hernia, it is usually found the best surgery to remove the herniated parts. The incisions are made as for ordinary hernia, and the appendages are removed according to the principles laid down. Mr. Lawson advises that the divided end of the Fallopian tube be fixed in the wound by a suture: this procedure seems to be no more necessary in operating here than by abdominal section. In most cases there will be found adhesions fixing the herniated organs in the sac. Hulke has operated upon a case in which one corner of a bifid uterus lay in the sac with the appendages, and "the inguinal portion of the uterus was invested by peritoneum, which passed directly into that of the hernial sac, and thus fixed the organ *in situ*." In herniated ovaries that are easily reducible there is danger of their slipping into the abdomen during operation: to avoid the risk of this, it is suggested that a needle be passed behind the ovary to fix it—a suggestion which is open to the objection that bowel may be pierced. In some cases, where the hernia only occasionally takes place, it may be most expedient to remove them by abdominal section.

*Ligature instead of Removal.*—In cases where removal is extremely difficult, or impossible, the proposal of Professor Simpson, to strangulate the blood supply by ligation, is worthy of a trial. In his hands, and in the hands of Leopold of Leipzig, and others, it has done good. Dr. Geza v. Antal, of Buda-Pest (*Centralbl. f. Gynäk.*, 1882, No. 30), urges atrophic ligature of the ovaries, not only for uterine fibroids, but for uterine versions or flexions, ovarian displacements, and other conditions. More than one writer has suggested that the whole proceeding of removal of the appendages does good entirely by cutting off blood supply.

*Removal by the Vagina* has now practically been abandoned, and need not be described. I have once operated in this way with success.

*Progress after Operation.*—The progress after operation in all essential particulars so closely resembles that seen after ordinary ovariotomy, that it need not be described. Two peculiarities may be mentioned—pain and uterine hæmorrhage.

After removal of the appendages there is usually, for a day or two, considerable pain in the hypogastrium, far more than is seen after removal of cystomata. We ought to be chary of having recourse at once to opium. Flatulence and sickness are so frequent sequences of opiates, however administered, that it is always wise to postpone their administration up to the limits of the patient's endurance. The pain soon passes off: it rarely continues over the second day. Battey used to divide the pedicle by ecraseur, to try and do away with this pain: statements as to the success of his practice are not published. If there is restlessness or jactitation, opium is specially indicated.

On the second, third, or fourth day after operation we may expect bleeding from the uterus to take place. The bleeding is usually considerable in amount, and may continue over four or five days. It is in no way harmful; indeed, it is usually accompanied by amelioration of the subjective symptoms. Some surgeons consider it part of the cure in the operation for myoma. It requires no treatment, and need cause no anxiety.

Treatment is advanced by casting off error almost as much as by introducing improvements. The hereditary fallacies in the treatment of abdominal operations are ice, milk, and opium : ice to allay thirst, milk as a food, and opium to treat peritonitis. Ice does not allay thirst; as cold water it lies unabsorbed in the stomach, to be ultimately rejected. For ice substitute hot water, or hot toast and water, or weak hot tea, or solution of beef essence, by the mouth; or a large water enema by the rectum. Milk is a bad food in abdominal cases; it causes flatulence—a most baneful result, it is not easily digested, and it is rarely acceptable to the patient. Peptonised, it is less objectionable. In these cases peritonitis is better treated by a saline purge than by opium. A purge relieves engorgement of the intestinal vessels, carries off flatulence, and diminishes fever; opium aggravates these symptoms, while it aggravates the conditions which cause them. The rectum tube and the turpentine enema, or the simple hot-water enema, will relieve flatulent distension more satisfactorily than any drug or any combination of drugs.

Finally, I would never confine the patient to the supine posture. If the parietes are well supported by strapping, the clumsy obstetric binder may be abolished, and the patient may from the day of operation be permitted to assume any position, short of the erect or the sitting, which is most comfortable to herself. A patient, restless and complaining of backache while in the supine position, will often drop off to sleep at once when placed on the side.

The time-honoured observance of catheterism every four or six hours is also a mistake. In most cases we may wait till the patient asks to be relieved: this may not be for twelve or more hours, as the secretion of urine is usually scanty for the first day or two after an abdominal operation. As soon as she can pass water, she may be permitted to do so.

These are commonplace and routine practices. I honestly believe that to let the patient alone as much as possible is the best practice. In nine cases out of ten the nurse can treat the patient as well as the surgeon; in the tenth, however, prompt and decisive measures may have to be adopted: such as drainage, opening and washing out the cavity, free purgation, artificial emesis; and it is here that skill, judgment and courage, more perhaps than in any other department of surgery, can record their triumphs.

*Remote Effects of the Operation.*—The individual results of the operation as performed for specific disease have already been described, and the general results so far as they affect the feminine attributes have been sufficiently discussed. It remains shortly to give an account of the behaviour of cases, with particular reference to the uterine functions and with general reference to bodily health as observed for a year or more after operation. Each disease has its own record, and all diseases have points in common.

For inflammatory disease, which is perhaps the most satisfactory source of operation, the record is broadly as follows:—At the end of a fortnight or three weeks the patient will probably express herself as feeling peculiarly well, and will be anxious to get up. When she has been getting about for a week or so, and at the time when menstruation is due, she will probably complain of backache and feelings of weight in the hypogastrium, and possibly her spirits may become a little depressed. Most probably there will be molimina, but no menstrual flow. During the next month slight backache, with some general weakness, may be complained of; and somewhere near the next period these symptoms may be aggravated. These cases do not get quite well very quickly as a rule; and this, of course, is not to be expected, considering their previously prolonged illness. In from three to six months perfect recovery may be expected. But all the old pain will have disappeared; and this alone, even in the most tedious cases, the patient will say was more than enough to justify operation. Regular menstruation is very rare, irregular and slight bleedings are more common; neither is likely to continue over more than six months. In a considerable majority amenorrhœa, immediate and permanent, follows the menorrhagia which occurs a few days after operation.

In myoma the result is very variable as to subsequent course. There occurs the ordinary metrorrhagia immediately after operation. Thereafter we may expect complete arrest of menstruation, with at first cessation in the growth of the tumours, and then slow but steady diminution in size. This shrinking is by no means confined to small tumours, and it is rarely possible to foretell what tumours will shrink and what will not shrink.

There is one variety of myoma—the soft, œdematous—which, Tait tells us, goes on growing after removal of the appendages, and which can be treated only by hysterectomy. I can find no definite information as to the utility of removal of the appendages after the period of the menopause has passed; and it would be dangerous, considering how little we really know of their functions even when supposed to be physiologically inert, to speculate as to the possible benefits to be derived from the proceeding. A tumour may not show signs of diminution in size for several months, when it may begin to decrease in bulk over one or two years, and then reach dimensions which are stationary.

For the neuroses, the results are at once highly encouraging and deeply disappointing. The balance, however, lies largely on the side of encouragement. The failures have been chiefly in cases of epilepsy; and here, no doubt, errors arising from an improper selection of cases have been most numerous. In favourable cases we are not to expect complete and perfect cure from the beginning. For the first few weeks there may be a complete cessation of the abnormal nerve phenomena; at the time of the next period there may be a few fits; then in diminishing numbers a few more fits, lasting over several months. Perfect recovery must not be counted upon within six months at least; and this recovery must be encouraged by strict attention to regimen and surroundings.

In concluding these remarks upon the operative details, I cannot refrain from expressing my belief that the operation has suffered at the hands of careless diagnosticians and inexperienced operators. The average mortality is high—over ten per cent.—simply because the single operations of inexperienced surgeons have to be included; and the results are not so perfect as they might be, because unsuitable cases are submitted to operation by men who are not sufficiently trained in this department of practice. *The* mortality is the best attained mortality: in the hands of one surgeon it is less than three per cent. Removal of the uterine appendages is not to be undertaken with a light heart. In ten cases which the writer has operated upon, he has had to contend with difficulties greater than in fifty ovariotomies. And some of these cases he could not have finished had he not had some experience in abdominal surgery. The dead-house is a valuable source of experience. There one may practically learn the lie of the appendages, how to find them, and how to remove them when healthy; and this is valuable tuition towards their removal in disease. Without such experience in the living or the dead, no surgeon ought to undertake removal of the uterine appendages. If he does, every tenth case will die, and every eleventh case he will fail to complete: doing so, he fails to do the best for his patient, and he brings his art into discredit.

### BIBLIOGRAPHY.

INFLAMMATION AND ABSCESS OF THE OVARIES.

TAIT.—*Diseases of the Ovaries.* 4th Ed. 1883. P. 87.
COURTY.—*Diseases of the Uterus.* (Trans. by A. McLaren.) 1882. P. 510.
SCHRŒDER.—*Diseases of the Female Sexual Organs.* (Ziemssen's Cyc.) 1875. P. 351.
TILT.—*Diseases of Menstruation and Ovarian Inflammation.* 1850.
GALLARD.—De l'ovarite aiguë et de la congestion pelvienne. *France Med.* 1884. i., p. 614.
LETULLE.—Ovarite suppurée . . . pelvi-peritonite . . . mort. *Ann. de Gynéc.* Par., 1884. xxii., p. 442.
ALEXANDER.—On Removal of the Ut. App. for Infl. Disease. *Med. Press and Circ.* 1884. xxxviii., p. 175.
TILT.—Diagnosis of Subacute Ovaritis. *Amer. Med. Journ.* Vol. lxvii.
BIGELOW.—A Case of Oöphoritis. *Cincin. Lancet and Clinic.* 1884. xxii., p. 377.
HAWKINS.—Removal of Ovaries and Fallopian Tubes for Ovaritis. *Denver Med. Times.* 1883-4. iii., p. 289.
IMLACH.—Specimens of Infl. Disease of Ovaries, &c., from 21 women. *Liverpool Med.-Chir. Journ.* 1885. v., p. 221.
RACIBORSKY.—*Gaz. des hôp.* Nov., 1856.
SCHULTZE.—*Jenaische Zeit. f. Med. u. Nat.* i. 1864. P. 279.
BOUVERET.—*Ann. de Gynéc.* iv., p. 427.
VERJUS.—*Thèses de Paris.* 1884. (Abscess bursting into peritoneum, causing death.)
PUECH.—*Gaz. des hôp.* 1860. P. 517. (Abscess bursting into peritoneum.)
BOURNAND.—De l'ovarite blenorrhagique. Par., 1847.
GAILARD.—*Gaz. des. hôp.* July–October, 1869.
DUNCAN.—*Ed. Med. Journ.* Sept., 1871.
SLAVJANSKY.—*Arch. f. Gynaek.* Vol. iii., p. 183.
LUSK.—Ovaritis with Abscess. *Amer. Journ. Obstet.* Jan., 1880.
IMLACH.—Ovarian Abscess and Pyosalpinx. *Liverpool Med.-Chir. Journ.* Jan., 1886.
MACDONALD.—Gonorrhœal Ovaritis. *Edin. Med. Journ.* Dec., 1885.

DISPLACEMENTS OF THE OVARY.

BARNES.—On Hernia of the Ovary. *Amer. Journ. Obstet.* Jan., 1883.
DENEUX.—Sur la hernie de l'ovaire. Par., 1813.
POTT.—Hernia of Ovaries successfully operated upon. *Works.* Vol. ii. (Occurred in 1756.)
WERTH.—Doppelseitige Hernia Ovarialis inguin. *Arch. f. Gynaek.* v., p. 11.
LOUMAIGNE.—De la hernie de l'ovaire. Par., 1869.
WEINLECHNER and BALLERAY.—Ovarial-hernie. *Centralbl. f. Chir.* v., p. 5. 1877.
CHAMBERS.—Congenital double Inguino-Ovarian Hernia. *Lond. Obstet. Journ.* Dec., 1879.
PUECH.—Des hernies de l'ovaire. *Gaz. Obstét. de Par.* 1875.
JONES (W. M.)—Congenital Hernia of both Ovaries. *Brit. Med. Journ.* Sept., 1877.
MULERT.—*Journ f. Chirurg.* 1850. ix., p. 3.
ENGLISCH.—*Medicinische Jahrbücher.* 1871. P. 335.
McCLUER.—*Amer. Journ. Obstet.* vi., p. 613. Operation.
NEBOUX.—*Bull. de Thérap.* Avril, 1845. Operation.
LOEPER.—*Monatsschr. f. Geb.* B. 28, p. 453. Operation.
LASSUS.—*Path. Chir.* Par., 1806. ii., p. 98. Operation.

GUERSANT.—*Bull. de Thérap.* 28. 1865. Two cases. Operation.
HOLMES.—*Lancet.* Jan., 1884. Operation.
MEADOWS.—*Trans. Lond. Obstet. Soc.* iii., p. 438. Operation.
CLEVELAND.—Adhäsion und Prolapsus des Ovarien. *Schmidt's Jahrbuch* v., 156.
MUNDÉ.—Prolapsus of the Ovaries. *Med. Times and Gaz.* Jan., 1880.
STOCKS.—Ovarien Prolaps. *Centralbl. f. Chir.* v., 1.
ATTHILL.—Prolapse of Ovaries. *Med Press and Circ.* Dec. 10th, 1880.
SKENE.—Prolapse of Ovaries. *Amer. Journ. Obstet.* April, 1879.
GOODELL.—Prolapse of Ovaries. *Lessons in Gynæcology.* Phila., 1880.
HUNTER.—Prolapsus of the Ovary. *Phila. Med. Times.* 1883-4. xiv., p. 559.
IUTZE.—Displacement of Left Ovary. Battey's Operation. Death. *Amer. Pract.* 1883. xxviii., p. 257.
HEYWOOD SMITH.—Hernia of the Ovary. *Brit. Gynæc. Journ.* Nov., 1885.

CYSTIC AND CIRRHOTIC OVARIES.

TAIT.—*Diseases of Ovaries.* 4th Ed.
JACOBI.—Cystic Ovaries. Battey's Operation. *New York Med. Rec.* 1884. xxx., p. 705.
WILL.—Cystic Degeneration of the Ovary. Battey's Operation. Death. *Peoria Med. Monthly.* 1883-4. iv., p. 419.
BROWN.—Cystic Ovaries removed by Laparotomy. Hydrosalpinx. *New York Med. Rec.* 1884. xxv., p. 195.
BERAUD.—(Variolous ovaritis.) *Archiv. Gén. de Med.* 1859. xiii., p. 588.
WALLACE.—Bilateral Ovarian Cystic Degeneration. Oper. Rec. *Lancet.* 1884. i., p. 1027.

DISEASES OF THE FALLOPIAN TUBES.

TAIT.—*Diseases of Ovaries.* 4th Ed.
*Tr. Med.-Chir. Soc. Edin.* 1883-4. iii., p. 36.
*Trans. Obstet. Soc. Lond.* 1884. Pp. 111, 138, 234.
*New York Med. Journ.* 1884. xl., p. 421.
*Med. Times and Gaz.* 1884. ii., p. 318.
*Edin. Med. Journ.* Sept., 1885.
*Brit. Gynæc. Journ.* July, 1885, and Nov., 1885.
SIMPSON.—Hæmato-salpinx. Case. Tait's Operation. *Edin. Med. Journ.* 1884. xxx., p. 447.
BROSSARD.—Tubo-ovarite suppurée avec péritonite pelvienne circonscrite. *Ann. de Gynéc.* Par., 1885. xxiv., p. 117.
CHAPMAN.—Extreme Cystic Dilatation of the Fallopian Tubes. Abdominal Section. Recovery. *Tr. Edin. Obst. Soc.* 1883-4. ix., p. 199. *Edin. Med. Journ.* 1884-5. xxx., p. 204.
SIMPSON.—Hæmato-salpinx. Tait's Operation. *Trans. Edin. Obstet. Soc.* 1883-4. ix., p. 182.
WYLIE.—Cases of Salpingitis. Results of Operations. *New York Med. Rec.* 1885. xxvii., pp. 85, 161.
Diseases of the Fallopian Tubes. *Boston Med. and Sur. Journ.* 1886. cxii., p. 109. *Amer. Journ. Obstet.* vi., p. 43.
FERGUSON.—The Diagnostic Value of Ciliated Columnar Epithelium in cases of Hydrosalpinx and Pyosalpinx. *New York Med. Journ.* 1885. xli., p. 211.
WIEDOW.—Zur operativen behandlung der pyosalpinx. *Centralbl. f. Gynäk.* Leipz., 1885. ix., p. 145.
PORTER.—Report on a case of Pyosalpinx. *Phila. Med. News.* 1885. xlvi., p. 362.
CHAMBERS.—Hydro-salpinx. *New York Med. Rec.* 1885. xxvii., p. 273.

ZEISS.—Pyo- und Hämato-salpinx. *Centralbl. f. Gynäk.* Leipz. 1885. vii., p. 745.

HORNE.—Case of Salpingo-oöphorectomy for Pyosalpinx. *Dublin J. Med. Sc.* 1884. lxxvii., p. 347.

KLOB.—*Path. Anat. d. Weibl. Sexualorgane.* P. 288.

HENNIG.—*Der Katarrh der inneren Weiblechen Geschlechtstheile.* 2nd Ed.

FÖRSTER.—*Wiener Med. Woch.* 1859. Nos. 44 and 45.

WAGNER.—*Monatsschr. für Geb.* Vol. xiv., p. 436.

VON DESSANER.—*Ibid.* Vol. xxvii., p. 60.

GOODELL.—Pyosalpinx and Hydrosalpinx. *Journ. Amer. Med. Asso.* Chicago, 1884. iii., p. 24.

MARTIN.—Successful Operation for Pyosalpinx. *Boston Med. and Surg. Journ.* 1884. cxi., p. 132.

MACDONALD.—Two Cases of Salpingo-oöphorectomy. *Ed. Med. Journ.* 1884-5. xxx., p. 97.

LEDIARD.—Pyosalpinx. Abdominal Section. Recovery. *Lancet.* 1884. ii., p. 493.

AVELING.—A Case of Double Pyosalpinx. *Brit. Gynæc. Journ.* July, 1885.

SANGER.—Gonorrhœal Disease of the Uterine Appendages, and the Operative Treatment. *Abst. in Amer. Journ. of Med. Sc.* Jan., 1885. P. 295.

OPERATIONS FOR CAUSES RESIDING IN UTERUS.

WERNER.—Battey's Operation performed in a case of Malformation of the Genital Organs. *Am. Journ. Obstet.* 1884. xvii., p. 144.

BATTEY.—*Trans. Internat. Med. Congress,* 1881. iv., p. 280.

TAIT.—Post-mortem Examination on case where Appendages were removed for Myoma three years before death. *Med. Times and Gaz.* 1884. ii., p. 147.

The Modern Treatment of Uterine Myoma. *Brit. Med. Journ.* Aug., 1885.

MENZEL.—Castrationen bei Ovarialprolaps. Uterus-fibrom. Retroflexio uteri mit Descensio Ovariorum. *Archiv. f. Gynäk.* Bd. xxvi. Hft. i.

WIEDOW.—(For Fibroids.) *Archiv. f. Gynäk.* Bd. i. 1885. p. 299.

DUPLAY.—De l'ablation des ovaires dans le traitement des fibro-myomes, &c. *Archiv. Gén. de Méd.* July, 1885.

Discussion on the Treatment of Uterine Myoma. *Brit. Gynaec. Journ.* July and November, 1885.

OVARIAN NEUROSES.

BIRCHER.—Die Castration bei Ovarial-neuralgie und Hysterie. *Cor. Bl. f. Schweiz. Aerzte.* 1884. xiv., pp. 447, 470.

MONTGOMERY.—Removal of Appendages for Menstrual Epilepsy. *Phila. Med. News,* 1884, xiv., 584; and *Amer. Journ. Obstet.*, 1885, xviii., 152.

HEGAR.—Der Zusammenhang der Geschlechtskrankherten mit Nervosen Leiden und die Castration bei Neurosen. Stuttgart, 1885, and *Centralbl. f. Gynäk.* 1884, xxxviii., p. 593.

BROOKS.—Castration for Hystero-epilepsy. *Phila. Med. and Surg. Rep.* 1885. iii., p. 230.

GOODELL.—Removal of Ovaries for Confirmed Masturbation. *Phila. Med. Times.* 1883-4. xiv., p. 230.

SIMS.—Battey's Operation in Epileptoid Affections. *N. Y. Med. Rec.*, June 5, 1880.

PALLEN.—True import of Oöphorectomy in Epilepsy. *N. Y. Med. Rec.* June 5, 1880.

TERRIES.—Sur l'influence que peut avoir l'ovariotomie sur les manifestations hysterique.—*Bull. et Mem. Soc. de Chir. de Paris.* 1884. x., p. 243.
HOMANS.—Hysteria as affected by Removal of the Ovaries. *Boston Med. and Surg. Journ.* 1884. cx., p. 542.
WALTON.—Hysteria as affected by Removal of the Ovaries. *Ibid.*, p. 529.
LANDAU AND REMAK.—Ein Fall von Ovariotomie bei hysterischer Hernia. nästhesie; Klinischer Beitrag zur Ovarie- und Castrations-Frage-*Ztschr. f. Klin. Med.* 1883. vi., p. 437.
MUNDÉ.—Removal of Appendages followed by disappearance of symptoms of Spinal Atrophy. *Amer. Journal Obstet.* 1884. xvii., p. 1162.
GEISSLER.—Hyperaesthesie des Ovarium. *Schmidt's Jahrbuch.* v., p. 76.
SCHMALFUSS.—Zur Castration bei Neurosen. *Archiv. f. Gynäk.* xxvi. i. p. 1.
LEPPMANN.—Castration bei Epilepsie und Hystero-Epilepsie. *Ibid.*
OHR.—Genito-Reflex Neuroses in the Female. *Amer. Journ. Obstet.*, 1883. xvi., p. 50 *et seq.*
CARSTENS.—Three Cases of Battey's Operation. *Ibid*, p. 266.

GENERAL CASES.

IMLACH.—Six Cases of successful Removal of the Uterine Appendages. *Liverpool Med.-Chir. Journ.* 1884. iv., p. 392; also *ibid*, Jan., 1886.
GRAY.—A Case of Battey's Operation. *Atlanta Med. and Surg. Journ.* 1884. i., 321.
MUNDÉ.—Three successful Cases of Oöphorectomy. *New Eng. Med. Monthly*, 1883-4. iii., p. 549.
DAWSON.—Two Cases of Oöphorectomy. *Amer. Journ. Obstet.* 1884. xvii., p. 964.
GOODELL.—Two Cases of Oöphorectomy. *Phila. Med. News.* 1884. xiv., p. 468; and *Amer. Journ. Obstet.* 1884. xvii., p. 1186.
VAN ERNAN.—Four Cases of Oöphorectomy. *Kansas City Med. Rec.* 1884. i., p. 95.
FEHLING.—Zehn Castrationen, &c. *Archiv. f. Gynaek.* Berl., 1883-4. xxii., p. 291.
HERMANCE.—Ovarian Dysmenorrhœa: Oöphorectomy. *N.Y. Med. Record.* 1884. xxv., p. 430.
JACKSON.—Double Oöphorectomy for Dysmenorrhœa, &c. *Weekly Med. Review.* Chicago, 1884, ix., p. 290.
THALLON.—The Battey-Tait Operation. *N. Y. Arch. Med.* 1884. xi., p. 180.
JACOBI.—Salpingo-Oöphorectomy. *N. Y. Med. Journ.* 1884. xxxix., p. 673.
THOMAS (T. G.)—Two Cases of Extirpation of the Ovaries. *Med. Chron.* Baltimore, 1883-4. ii., p. 161.
CHÉREAU.—Memoire pour servir à l'étude des maladies des ovaires. Par., 1884.
ENGELMANN.—Case of Battey's Operation. *Boston Med. and Surg. Journ.* May 13, 1880.
SAVAGE.—On Oöphorectomy. *Lond. Obstet. Journ.*, May, 1880; and *Trans Internat. Med. Cong.* 1881. Vol. iv.
BATTEY.—Summary of 15 Cases of Battey's Operation. *Brit. Med. Journ.* April 3, 1880.
EWENS.—Two Cases of Oöphorectomy. *Brit. Med. Journ.* Jan. 31, 1880.
THOMAS.—Normal Ovariotomy. *Amer. Med. Journ.* Vol. lxvii.
LUTAUD.—Ovariotomie Normale. *Arch. Gén. de Méd.* 1879. Vol. i.
MEADOWS.—Ovarian Menorrhagia. *Brit. Med. Journ.* July 12, 1879.
VERNEUIL and FERRIER.—Menstruation after Double Ovariotomy. *Lond. Obstet. Journ.* Vol. v.
DE SINÉTY.—Ovulation et Menstruation. *Ann. de Gynéc.* Vol. vii.
LOEWENTHAL.—Une nouvelle Théorie de la Menstruation. *Med. Pract.* Par., 1884, v., p. 577; and *Semaine Med.* Par., 1884, iv., p. 461.

HEGAR.—Die Castration der Frauen. *Samml. Klin. Vortr.* Leipz., 1878. Nos. 136-8.
BAKER (W. H.).—The Use and Abuse of Battey's and Tait's Operations. *Boston Med. and Surg. Journ.* 1885.
SIMPSON.—Ligature of Broad Ligaments instead of Battey's Operation. *Edin. Med. Journ.* 1884–5. xxx., p. 444.
GARDNER.—Ovaries and Fallopian Tubes removed, &c. *Canada Med. Rec.* 1882–3. xi., p, 242.
JACKSON.—A Contribution to the Relations of Ovulation and Menstruation. *Journ. Amer. Med. Ass.* Chicago, 1884. And Reprint.
GALLARD.—Physiologie de la Menstruation. *Compt. rend.* 1883–4. xii., p. 713.
KYLE.—Oöphorectomy. *Louisville Med. News.* 1885. xix., p. 129.
RABAGLIATI.—Two Cases of Double Oöphorectomy. *Lancet.* 1884. ii., p. 1091.
CHUNN.—Removal of Uterine Appendages. *Maryland Med. Journ.* 1883–4. x., p. 526.
HOOPER.—The Morbid Anatomy of the Human Uterus and its Appendages Lond., 1832. (Specially for diseases of the Fallopian tubes.)
JOHNSON (J. T.)—Four Cases of Oöphorectomy, with remarks. *N. Y. Med. Journ.* Sept. 26th, 1885.
PARISH.—Removal of the Uterine Appendages. *Phila. Med. Times.* Jan. 23rd, 1886.
TAYLOR, (J. W.)—Report on Abdominal Surgery. *Birm. Med. Rev.* Sept., 1885.
KEEN.—Three Cases of Tait's Operation. *Phila. Med. Times.* xvi., p. 343.

---

### APPENDIX OF CASES.

Some of these cases—as those of hystero-epilepsy—are of sufficient interest to be worthy of publication in detail: when a sufficient time has elapsed to make it certain that the full effects of the operation have been secured, they will be published. All the cases recovered.

1. *Hystero-epilepsy.*—Nine years. Pyo- and hæmato-salpinx; ovaritis. General matting of appendages by old dense adhesions. Operation very difficult. Free hæmorrhage; broad ligament torn from uterus; appendages cut away at a distance from the surface, and six ligatures placed. Drainage.

2. *Hystero-epilepsy.*—Ten years. One ovary enlarged, cystic and sessile on fundus uteri; the other lying in Douglas's pouch, and slightly adherent. Operation easy.

3. *Prolapse and Chronic Inflammation.*—Appendages fixed in the retro-uterine *cul-de-sac* by old and very strong adhesions. Hæmato-salpinx and ovaritis. Operation very difficult. Free bleeding from raw surfaces, and great injury done to broad ligaments. Drainage. Symptoms of intestinal obstruction on fourth day, with stercoraceous vomiting. Abdomen reopened and washed out.

4. *Prolapse.*—One ovary prolapsed, enlarged and adherent in Douglas's pouch. Removed by vaginal incision.

5. *Ovarian Abscess.*—One ovary, as large as a pigeon's egg, contained abscess; the other, cirrhotic. Both prolapsed and adherent.

6. *Displacement and Adhesions.*—Left ovary attached by one surface to rectum; by the other, to the tip of the vermiform appendix; much enlarged, and containing two large blood-clots. Right ovary very small and cirrhosed. Left Fallopian tube contained much black fluid blood.

7. *Cystic and Cirrhotic Ovaries, with fine Adhesions.*—Ovaries slightly enlarged; tissue consisting of dense, almost gristly, material surrounding several cysts. The lining membrane of one cyst contained inorganic muscle. Numerous long thread-like adhesions to bowels and broad ligaments. Hæmato-salpinx.

8. *Bleeding Myoma.*—Both ovaries enlarged, and studded with numerous small cysts; tubes normal.

9. *Double Hydro-salpinx.*—One tube contained about six ounces of fluid, full of cholesterine; the other, about two ounces of clear yellow fluid. Ovaries left. Menopause.

10. *Hæmato-salpinx.*—More than a pint of black treacly blood in one tube, universally adherent. Ovary embedded in cyst wall. Appendages on opposite side not removed.

11. *Pyo-salpinx.*—Globular tumour, size of orange, with very thick walls, containing putrid pus, shelled out of capsule of inflammatory tissue. Cyst in broad ligament of opposite side. Drainage. Ovary enlarged, but not diseased.

12. *Pyo-salpinx.*—Thick-walled abscess; universally adherent walls; contents excessively putrid, more than a pint of pus, and some gas. Patient much exhausted with hectic and bronchitis. Tumour removed. Drainage and daily irrigation of abdomen for three weeks. Large sloughs removed from abdomen. Intestinal fistula spontaneously becoming cured. Ovary found in cyst wall.

